# Bilharziasis

**Published:** 1908-03-28

**Authors:** F. M. Sandwith


### BILHARZIASIS.

By F. M. SANDWITH, M.D., F.R.C.P.

Abstract of a lecture delivered at the Medical Graduates' College and Polyclinic on March 16, 1908.

Bilharziasis is a convenient term used for the invasion of the human body by the worm known as the *Schistosomum* or *Distomum hæmatobium*. The worm was discovered by one of my predecessors in Egypt, Bilharz, and is consequently often called by his name.

It is, as you probably all know, not the actual worm that does the harm, but its eggs. As you also know, this is a trematode worm, and the name Distomum indicates that it has two mouths. The male worm carries the female in the gynæcophoric canal. Besides the two mouths, there are to be noticed small tubercles all over the body of the male. It is by means of these that he makes his way along the blood-vessels: the female, having none, cannot move, and must, therefore, be carried by the male. Although the female is actually longer than the male—about 20 mm., as compared with 12 to 14—she is capable of concealing herself entirely in the gynæcophoric canal, for she is very much thinner and can double herself up.

The eggs are of two kinds, the lateral-spined and the terminal-spined, provided with a rudder wherewith to steer their passage. There has been some controversy as to whether the two varieties of egg are laid by the same individual, or even by the same species. The most widely-accepted view is that of Looss, that the lateral-spined egg is the product of an immature female, and that such a very young mother is unable to provide a shell sufficient to enclose all the egg-contents: consequently there is a bulging produced which alters the position of the spine. Later on, when the female has had more practice, she lays more perfect and terminal-spined eggs. Some do not accept this hypothesis, believing that there are two different kinds of worm; I do not myself think there is very much in favour of this view.

The terminal-spined eggs are always associated with the urinary tract, whereas the others are often to be found in the alimentary system, the liver, lung, rectum, intestine, and other non-urinary places. These eggs are deposited in the urine in very large numbers, and a normal egg thus passed in bloody urine will not develop further, nor will it when milk is added to the urine. But if it is put into pure clean water at a warm temperature it will develop into a miracidium. This is an embryo worm covered all over with cilia: it has a mouth leading to an œsophagus and to excretory ducts; but the greater part of its body consists of germinal cells. There are also two glands, one on each side of the œsophagus, which open externally one on each side of the mouth. The function of these is not known, but it has been thought possible that they may secrete something which enables the miracidium to penetrate the skin.

The process of development into the adult worm has not yet been traced. It is known that the miracidium does not survive under the most favourable circumstances longer than 36 hours, and hence it is supposed that there is an intermediate host, possibly a mollusc. But despite most careful search, no such host has yet been found.

There are three ways possible by which the worm may enter the body. The most obvious is by the mouth, but there are two or three objections to this, foremost among which is the fact that the miracidium dies at once in gastric juice only a quarter of the strength of that present in the healthy human stomach. It has also been proved by experiment that the gastric juice of monkeys is immediately fatal to the miracidium when it is introduced into the stomach of one of those animals. Then the urethra and rectum have been suggested, but there is really no evidence in support of this view, and in the organs of those who are the subjects of the disease the youngest worms are found in the liver, not in the rectum or the urinary tract. There is a story that in Siam the natives believe that the worm enters by the penis, and a South African custom has also been held to show the same belief: with regard to the latter I am told, however, that the natives always remove the protective apparatus when crossing a stream, which upsets this idea, and I am quite prepared to hear from Siam that the story is equally mythical there. The third possibility is that the embryo enters by the skin: as we now know that the Ankylostomum duodenale and other worms enter that way, it seems less improbable than would have been thought a few years ago. The proof is still wanting, for no one has yet felt justified in putting it to the test of experiment.

The two countries more especially concerned with Bilharziasis are Egypt and South Africa, though it is also known in Tunis, Mauritius, the Congo, Cyprus, Uganda, and other places. There is every difference between the disease in the north and in the south of Africa. In the former it is a very severe disease and frequently causes fatal complications, though I know of no reason for its greatly lessened virulence in South Africa. The disease is not so common in women as in men, but it is not really so rare in them as some of the text-books would lead one to believe. The bladder is the organ usually most infiltrated, and the result in an advanced case is a thickening of the wall which may look to the naked eye very like cancer, and, indeed, can only be recognised microscopically. In the liver it may cause cirrhosis, and should be added to the list of the agents which may produce that condition. In the rectum and colon papillomata are formed, which may slough away. Pus may run from the urethra, once supposed to be due to gonorrhœa, but now known to be often due simply to the Bilharzia. Cystitis is the rule in the bladder, and large numbers of calculi are formed there.

The incubation period is from two to four months. The parasite may be present for six or more months without giving rise to any symptoms. Natives do not trouble themselves at all about the early symptoms, regarding them as no more unusual or serious than we do bruises after a football match. One of the earliest symptoms is hæmaturia: the blood does not come with the urine, but immediately after, due to the final contraction of the bladder-wall forcing the spine of an egg into a capillary. It is in this terminal teaspoonful of blood-infiltrated urine that you may expect to find the eggs, and not in the rest of the urine. By examining it thus you will always find eggs in a case of bladder-infection. Many things increase the hæmorrhage—exercise, drinking, and so on.

There is no pain at the beginning of the disease, but there are one or two symptoms similar to those of vesical calculus, such as pricking at the end of the penis. After that come frequent micturition, lumbar aching, incontinence, and later on a septic cystitis when germs have obtained entrance into the bladder. Papillomata and sloughs cause severe tenesmus, and enlargement of the prostate adds to these troubles.

In a rectal case there is at first only a slight discharge of mucus, and the patient generally comes complaining of piles. Besides the straining, which is also an early symptom, there then ensues prolapse, and the patient thinks he has got dysentery; indeed, his medical man often thinks so too, for mucus, diarrhœa, tenesmus, and blood are, after all, the usual basis for such a diagnosis. These symptoms are due to infiltration of the mucous membrane near the sphincter, which is generally most affected. Then follow ulceration and pain, though the condition of the patient is not so miserable as that of him who is affected in the bladder. In the vagina large papillomatous masses are produced, and the uterus may also be affected. In the latter the appearances simulate epithelioma, and the uterus has been removed under that diagnosis.

The amount of blood lost is not enough to produce great anæmia. The usual blood-count is about 4,000,000 red corpuscles per c.m., and 80 per cent. of hæmoglobin. The eosinophils are increased, as might be expected: in a series of cases the average was found to be 16 per cent., but I have known them as high as 53 per cent.

Dangerous hæmorrhage from the bladder may so fill that organ with blood clot as to cause death from uræmia. A palpable tumour of the enlarged bladder may assist in the diagnosis of Bilharziasis.

Hydronephrosis and pyonephrosis often occur, and what used to be called surgical kidney is also seen: these are due to fibrosis or stone in the ureter producing stricture, and dilatation above it, after which comes a huge dilatation of the kidney itself. There is not usually any deposit of eggs in the kidney. Vesical calculi are common, and can be diagnosed by the sound. In the College of Surgeons Museum is one weighing 34 ounces, removed from a patient who lived for two and a half months after: the rapidity with which such stones form was shown by the removal of a second stone weighing 23 grains on the 25th day after the first operation.

Urinary fistulæ are common complications, and the most important are those in the floor of the urethra. Though they are very troublesome, they can be cured by patient and appropriate surgical treatment. Cancer is not very common, considering the enormous amount of irritation set up in the organs which harbour the parasites.

The worms themselves are found in the portal vein, and can be obtained post-mortem by putting a spoon into it and scooping out the blood. From one or two to several hundred may thus be found. If none are found in the portal vein in a suspected case, the liver should be cut into sections and pressed out in water. The spleen may contain eggs, and the broad liagment and the skin have also been known to harbour them.

The prognosis is important, even in this country, because many men invalided from Africa for this disease are to be found here. The usual view held by English physicians is, I think, unnecessarily gloomy. Unless a man is over 50, or is advanced case, he will certainly improve or get well altogether in a few years even with almost no treatment.

There are many suggested remedies which are no good: the only one which is of use is the liquid extract of male fern. Give it in 15-minim doses, but not for more than 14 days together. This drug actually reduces the number of eggs and the hæmorrhage, and also relieves the cystitis to some degree. Hæmostatic injections are positively bad. Salol, urotropin, and helmitol are all of use; and methylene blue is better still; it acts as an analgesic, but does not cure. Before I leave the question of Bilharziasis, which is almost confined to Africa, I should like just to remind you that the *Schistosomum japonicum* is an allied worm very similar in many ways to that of which I have been speaking.

